# Intra and intersession repeatability and reliability of dynamic parameters in pressure platform assessments on subjects with simulated leg length discrepancy. A cross-sectional research

**DOI:** 10.1590/1516-3180.2020.0791.R1.110321

**Published:** 2021-06-25

**Authors:** Héctor Pereiro-Buceta, César Calvo-Lobo, Ricardo Becerro-de-Bengoa-Vallejo, Marta Elena Losa-Iglesias, Carlos Romero-Morales, Daniel López-López, Eva-María Martínez-Jiménez

**Affiliations:** I MSc, DPM. Doctoral Student, Researcher, Health and Podiatry Group, Department of Health Sciences, Faculty of Nursing and Podiatry, Universidade da Coruña, Ferrol, Spain.; II PT, MSc, PhD. Senior Professor, Facultad de Enfermería, Fisioterapia y Podología, Universidad Complutense de Madrid. Madrid, Spain.; III RN, BSc, MLIS, DPM, DHL, PhD. Full Professor, Facultad de Enfermería, Fisioterapia y Podología, Universidad Complutense de Madrid. Madrid, Spain.; IV MSc, PhD, DPM. Full Professor, Faculty of Health Sciences, Universidad Rey Juan Carlos, Alcorcón, Spain.; V PT, MSc, PhD, Senior Lecturer, Faculty of Sport Sciences, Universidad Europea de Madrid, Villaviciosa de Odón, Madrid, Spain; VI MSc, PhD, DPM. Senior Lecturer and Researcher, Health and Podiatry Group, Department of Health Sciences, Faculty of Nursing and Podiatry, Universidade da Coruña, Ferrol, Spain.; VII PT, MSc, PhD, DPM. Assistant Professor, Facultad de Enfermería, Fisioterapia y Podología, Universidad Complutense de Madrid. Madrid, Spain.

**Keywords:** Leg length inequality, Gait, Equipment failure analysis, Pressure platform, Lower limbs, Reliability analysis

## Abstract

**BACKGROUND:**

Leg length discrepancy (LLD) may play a key role in exercise biomechanics. Although the Podoprint platform has been used in dynamic pressure studies, there are no data regarding the reliability and repeatability of dynamic measurements under simulated LLD conditions.

**OBJECTIVES:**

To determine the intra and intersession repeatability and reliability of dynamic parameters of the Podoprint pressure platform under simulated LLD conditions.

**DESIGN AND SETTING:**

Observational cross-sectional study at a public university.

**METHODS:**

Thirty-seven healthy volunteers participated in this study. LLD was simulated using ethyl vinyl acetate plantar lifts with heights of 5 mm, 10 mm, 15 mm and 20 mm located under the right shoe of each volunteer. The procedure was performed to capture the dynamic parameters of each participant under five different simulated LLD conditions. Stance time, mean pressure and peak pressure measurements were registered in three trials for each foot and each LLD level. Data were collected during two separate testing sessions, in order to establish intrasession and intersession reliability.

**RESULTS:**

The intraclass correlation coefficients (ICCs) for intrasession reliability ranged from 0.775 to 0.983 in the first session and from 0.860 to 0.985 in the second session. The ICCs for intersession reliability ranged from 0.909 to 0.990. Bland-Altman plots showed absence of systematic measurement errors.

**CONCLUSIONS:**

The results from this study indicate that the Podoprint platform is a reliable system for assessing dynamic parameters under simulated LLD conditions. Future studies should evaluate plantar pressures under LLD conditions, in association with exercise, biomechanics and musculoskeletal disorders.

## INTRODUCTION

Leg length discrepancy (LLD) is a situation in which the lower limbs have different lengths.^1^ LLD has been discussed in the clinical and research communities for decades. However, there is no consensus regarding many aspects of LLD, including its prevalence, the scope of its clinical significance, assessment of measurement methods and its impact on several neuromusculoskeletal alterations.^2^ LLD is estimated to affect 40-70% of the population and approximately 0.1% present inequality greater than 2 cm.^1^ Knutson conducted a meta-analysis on 573 subjects and concluded that only 10% of the population had equal lower-limb lengths.^3^

LLD can be classified as anatomical when the difference in length between the limbs can be measured directly in the tibias, femurs, or both; or it can be classified as functional when the discrepancy is caused by postural inaccuracies.^4^ LLD has been correlated with several pathological conditions, such as hip or knee osteoarthritis, due to inappropriate distribution of mechanical loads.^5^^,^^6^ Asymmetry in the kinetics of LLD-induced gait has also been linked to the etiology of plantar fasciitis,^7^ lower back pain^8^ and knee pain.^9^ Concretely, LLD may play a key role in skeletal malalignment disorders such as patellofemoral conditions.^10^ Asymmetries in the kinematics of human gait have been correlated with different degrees of anatomical LLD,^5^ such as pelvic drop and hip adduction in the stance phase.^11^^,^^12^ Additional studies have found flexion abnormalities in the sagittal plane of the hip, knee, ankle and first metatarsophalangeal joint.^11^^,^^13^^,^^14^^,^^15^ LLD also appears to be related to decreased load times in the shorter limb, reduced gait velocity, decreased stride length on the shorter leg and increased cadence.^16^ In addition, several studies have found that LLD produces asymmetrical pressure patterns.^17^^,^^18^

As described in the literature, there are two methods for studying LLD: evaluation of real LLD cases or simulation of LLD in healthy subjects, to assess the role of LLD regarding gait alterations. The first method has limitations because subjects with LLD commonly develop physical anomalies as a result of compensations and therefore cannot be considered to be pure LLD subjects. Moreover, LLD may frequently be associated with several disease processes that can also alter gait.^2^ To address these limitations, Betsch et al. designed a noninvasive method for simulating and studying LLD and its consequences on the musculoskeletal apparatus, using plantar lifts.^19^^,^^20^

Pedobarography is the study of foot-ground pressures. It has been used to study foot interactions with the ground and with posture,^21^ and to screen for plantar footprint alterations in healthy subjects that could lead to pathological conditions.^22^ Foot pressure measurement devices, essentially consisting of platforms and instrumented insoles, are used to quantify the static and dynamic parameters between the foot and floor; foot and shoe; and shoe and floor.^23^^,^^24^ These devices are generally intended for both clinical and research use.

Consequently, assessments on the reliability, validity and effectiveness of pressure systems are extremely relevant.^22^^,^^25^ A variety of plantar pressure devices are available, but one of the most commonly used devices in clinical podiatry is the Podoprint platform (Namrol Group, Barcelona, Spain). While this platform has been used in dynamic pressure and postural analysis studies,^24^^,^^26^^,^^27^ there are no data regarding the reliability and repeatability of dynamic measurements made using the platform. As previously mentioned, although a few studies have reported that LLD causes alterations to gait time and pressure patterns, the reliability and validity of the platform measurements have not, to the best of our knowledge, been addressed.^28^^,^^29^

## OBJECTIVE

Our aim was to assess the reliability and repeatability of the Podoprint platform for measuring dynamic plantar parameters obtained from healthy subjects under simulated LLD conditions. We hypothesized that the pressure platform could be accurately used to assess gait dynamic parameters, in order to study simulated LLD effects.

## METHODS

### Design and sample

Thirty-seven healthy volunteers (24 women and 13 men) aged 19 to 76 years participated in this study (Table 1). We used an observational cross-sectional research design in accordance with the criteria of Strengthening the Reporting of Observational Studies in Epidemiology (STROBE)^30^ and a nonrandom consecutive sampling technique. The inclusion criteria were that the subjects needed to be over 18 years of age; have a normal gait pattern;^31^ and have normal leg and foot joint ranges. Subjects were excluded if they reported having pain, anatomical or functional LLD,^32^ previous lower limb surgery, congenital or acquired deformities of the lower extremities upon clinical examination, or any other condition that might affect their gait.

**Table 1.** Descriptive data on the study participants showing demographics and anthropometric characteristics for the total sample and according to sex

Variable	Male n = 13	Female n = 24	Total (n = 37)
	mean ± SD (95% CI)	mean ± SD (95% CI)	mean ± SD (95% CI)
Age (years)	48.08 ± 17.38 (37.57-58.58)	42.17 ± 14.76 (35.93-48.40)	44.24 ± 15.75 (38.99-49.50)
Weight (kg)	73.38 ± 8.31 (68.36-78.40)	64.50 ± 12.95 (59.02-69.97)	67.62 ± 12.19 (63.55-71.68)
Height (cm)	174.46 ± 9.27 (168.85-180.06)	163.62 ± 8.54 (160.01-167.23)	167.43 ± 10.14 (164.05-170.81)
BMI (kg/m^2^)	24.18 ± 2.82 (22.47-25.89)	24.74 ± 5.32 (22.49-26.99)	24.54 ± 4.56 (23.02-26.07)
Foot size (EC)	42.23 ± 2.37 (40.79-43.66)	38 ± 1.37 (37.41-38.58)	39.48 ± 2.69 (38.58-40.38)

BMI = body mass index; SD = standard deviation; 95% CI = 95 percent confidence interval; EC = Europe countries.

The sample size was calculated using the GRANMO software, version 7.12, which was previously developed through a research program on inflammatory and cardiovascular disorders, at IMIM-Hospital del Mar, Barcelona, Spain.^33^ A study on the correlation between the dynamic parameters of gait and different degrees of simulated LLD was used as a reference.^28^ With a standard deviation (SD) of 4.34-3.48, 80% statistical power (β = 20%), a 95% confidence interval (α = 0.05) and two-tailed tests, it was calculated that 37 participants were required to detect a difference greater than or equal to 0.4 units. A SD of 0.86 and a loss to follow-up rate of 0% was assumed.

### Dynamic data collection

LLD was simulated using ethyl vinyl acetate plantar lifts of 70A Shore hardness and heights of 5 mm, 10 mm, 15 mm or 20 mm, which were attached with adhesive double-sided tape to the bottom of the volunteer´s own right shoe. This method mimics LLD by producing pelvic obliquity. In order to record the dynamic parameters, a Podoprint platform was integrated into the center of a flat 4.8 m walkway at ground level. The platform measured 610 mm x 580 mm with a thickness of 10 mm and a weight of 6.6 kg. It was composed of a self-calibrating system with 1600 10 mm x 10 mm resistive sensors.

Each subject was instructed to walk naturally while looking forward. The starting position was determined so that the footstep would coincide on the platform. Participants walked at a self-selected speed for all trials; however, speeds were within the limits considered to be normal cadence under laboratory conditions, which range from 81-138 steps per minute.^34^

The procedure was performed with the aim of capturing the dynamic parameters of each participant under five simulated LLD conditions with increasing amounts of plantar lift (0 mm, 5 mm, 10 mm, 15 mm and 20 mm). Stance time (ms), mean pressure (g/cm^2^), and peak pressure (g/cm^2^) measurements were recorded. These parameters were regarded as the ones most frequently used in functional foot appraisals under pathological conditions.^25^

Two testing sessions were conducted on seven separated days. Three trials for each foot and LLD level were collected per session. Before recording the dynamic data, all subjects completed three minutes of walking on the walkway to familiarize themselves with the platform and the plantar lifts. Four steps of each foot were registered per trial using the “multiple dynamic” mode of the platform, which automatically provides averaged parameters. The sampling rate was 100 Hz. The same researcher measured all subjects.

The data obtained from the platform were processed and stored using the manufacturer-specific software Podoprint for Windows, version 8.6.5 (Namrol Group, Barcelona, Spain), on a computer.

### Statistical analysis

All data were checked for normal distribution by means of the one-sample Kolmogorov-Smirnov test. Normally distributed data were presented as the mean and standard deviation.

Intrasession reliability was measured from three repeated trials for each simulated LLD condition and for each foot during the first and second testing sessions. The intraclass correlation coefficient (ICC) obtained using the (2,1) model (two-way random, single-measurement, absolute agreement ICC model) was calculated first, in order to analyze the reliability between trials.^35^ The coefficient of variation (CoV)^36^ was used to refer to the relationship between the size of the mean and the variability of each of the variables studied. The standard error of measurement (SEM) was expressed as a percentage of the mean (SEM%).^37^

In addition, the minimum detectable change (MDC) was calculated. This was defined as the magnitude of the value variation of each scale, below which change can be interpreted as inherent to the variability of the measurement method, without any real change to the clinical situation of the subject. MDC was calculated as a standardized mean (95% MDC).^38^^,^^39^

Intersession reliability was ascertained by retesting all subjects seven days after the first session. The average of the measurements for each session, for each subject and LLD condition, was used to calculate the ICC (2,1). For absolute comparison of the results obtained in the two sessions, CoV, SEM and MDC were expressed as percentages of the mean. The repeatability coefficient (RC) was also calculated as a percentage of the average values from the two measurement sessions, while the limits of agreement (LoA)^37^ were calculated to define the amount of variation that might be influencing the measurements. In addition, we used Bland-Altman plots to evaluate systematic measurement errors.

Paired Student’s t tests were used to determine systematic differences between the first and second sessions. From this, P-values were obtained, such that if P < 0.05, then there was a difference between the two variables. The IBM SPSS for Windows statistical package, version 22.0, was used for data analysis (SPSS, Inc., Chicago, Illinois, United States).

### Ethical considerations

The Research Ethics Committee of Universidad Rey Juan Carlos, Spain, issued the authorization certificate number 0904201907519, approved on May 22, 2019, for this study, which followed the ethical principles of the Helsinki declaration.^40^ All subjects signed an informed consent statement before participating in this study.

## RESULTS

### Intrasession reliability

Normative data (represented by mean ± SD) and reliability data (represented by ICC, CoV, SEM% and MDC%) for the first session are presented in Table 2. The ICC for intrasession reliability ranged from 0.775 to 0.983 and the SEM ranged from 0.059% to 1.095%. The CoV ranged from 0.322% to 2.474% and the MDC ranged from 0.051% to 3.037%.

**Table 2.** Repeatability of dynamic variables for each foot under simulated LLD conditions. First session

Variable	Mean ± SD	CoV (%)	ICC (2.1) (95% CI)	SEM%	95% MDC%
**0 mm of LLD**					
Stance time left (ms)	780.360 ± 7.131	0.913	0.898 (0.825-0.944)	0.291	0.808
Stance time right (ms)	772.613 ± 6.698	0.866	0.917 (0.857-0.954)	0.249	0.692
Mean pressure left (g/cm^2^)	763.027 ±11.271	1.447	0.915 (0.854-0.953)	0.43	0.156
Mean pressure right (g/cm^2^)	769.324 ± 7.263	0.944	0.892 (0.813-0.941)	0.31	0.111
Peak pressure left (g/cm^2^)	1449.297 ± 13.402	0.924	0.914 (0.852-0.953)	0.271	0.051
Peak pressure right (g/cm^2^)	1445.532 ± 17.208	1.19	0.931 (0.881-0.962)	0.312	0.866
**5 mm of LLD**					
Stance time left (ms)	778.396 ±13.756	1.767	0.873 (0.783-0.930)	0.628	1.743
Stance time right (ms)	770.351 ± 14.163	1.839	0.775 (0.613-0.876)	0.872	2.417
Mean pressure left (g/cm^2^)	776.240 ± 7.500	0.966	0.940 (0.896-0.967)	0.237	0.656
Mean pressure right (g/cm^2^)	761.991 ± 10.201	1.339	0.912 (0.849-0.952)	0.397	1.101
Peak pressure left (g/cm^2^)	1495.225 ± 12.651	0.846	0.906 (0.837-0.948)	0.259	0.719
Peak pressure right (g/cm^2^)	1365.090 ± 16.766	1.228	0.928 (0.877-0.961)	0.33	0.913
**10 mm of LLD**					
Stance time left (ms)	787.207 ± 6.588	0.837	0.973 (0.954-0.985)	0.138	0.381
Stance time right (ms)	793.063 ± 5.697	0.718	0.976 (0.959-0.987)	0.111	0.308
Mean pressure left (g/cm^2^)	780.432 ± 5.791	0.742	0.965 (0.940-0.981)	0.139	0.385
Mean pressure right (g/cm^2^)	741.081 ± 12.886	1.739	0.919 (0.861-0.956)	0.495	1.372
Peak pressure left (g/cm^2^)	1456.297 ± 9.645	0.662	0.909 (0.843-0.950)	0.2	0.554
Peak pressure right (g/cm^2^)	1274.694 ± 26.494	2.078	0.869 (0.774-0.928)	0.752	2.085
**15 mm of LLD**					
Stance time left (ms)	786.486 ± 7.819	0.994	0.983 (0.970-0.991)	0.13	0.359
Stance time right (ms)	798.378 ±7.206	0.903	0.980 (0.965-0.989)	0.128	0.354
Mean pressure left (g/cm^2^)	779.225 ± 2.512	0.322	0.966 (0.942-0.982)	0.059	0.165
Mean pressure right (g/cm^2^)	721.721 ± 5.353	0.742	0.922 (0.866-0.957)	0.207	0.574
Peak pressure left (g/cm^2^)	1451.928 ± 7.477	0.515	0.889 (0.807-0.939)	0.172	0.476
Peak pressure right (g/cm^2^)	1247.279 ± 22.891	1.835	0.778 (0.617-0.878)	0.865	2.397
**20 mm of LLD**					
Stance time left (ms)	788.288 ± 3.357	0.426	0.975 (0.956-0.986)	0.067	0.187
Stance time right (ms)	807.928 ± 4.092	0.506	0.976 (0.959-0.987)	0.078	0.217
Mean pressure left (g/cm^2^)	782.468 ± 3.751	0.479	0.964 (0.938-0.980)	0.091	0.252
Mean pressure right (g/cm^2^)	723.595 ± 2.566	0.355	0.894 (0.817-0.942)	0.115	0.32
Peak pressure left (g/cm^2^)	1446.685 ± 23.562	1.629	0.917 (0.857-0.954)	0.469	1.301
Peak pressure right (g/cm^2^)	1232.351 ± 30.494	2.474	0.804 (0.663-0.892)	1.095	3.037

SD = standard deviation; CoV = coefficient of variation; ICC = intraclass correlation coefficient; 95% CI = 95 percent confidence interval; SEM% = standard error of measurement percentage; MDC% = minimum detectable change percentage; LLD = leg length discrepancy.

Normative data and reliability data for the second session are shown in Table 3. The ICC for intrasession reliability ranged from 0.860 to 0.985 and the SEM ranged from 0.014% to 0.635%. The CoV ranged from 0.077% to 1.848% and the MDC ranged from 0.038% to 1.759%.

**Table 3.** Repeatability of dynamic variables for each foot under simulated LLD conditions. Second session

Variables	Mean ± SD	CoV (%)	ICC (2.1) (95% CI)	SEM%	MDC%
**0 mm of LLD**					
Stance time left (ms)	775.099 ± 0.595	0.077	0.968 (0.945-0.983)	0.014	0.038
Stance time right (ms)	771.712 ± 5.022	0.651	0.974 (0.955-0.986)	0.105	0.291
Mean pressure left (g/cm^2^)	776.775 ± 6.734	0.867	0.958 (0.929-0.977)	0.178	0.492
Mean pressure right (g/cm^2^)	786.541 ± 7.346	0.934	0.923 (0.867-0.958)	0.259	0.718
Peak pressure left (g/cm^2^)	1473.108 ± 9.370	0.636	0.923 (0.868-0.958)	0.177	0.489
Peak pressure right (g/cm^2^)	1476.541 ± 14.250	0.965	0.958 (0.927-0.977)	0.198	0.548
**5 mm of LLD**					
Stance time left (ms)	781.739 ± 11.497	1.471	0.860 (0.758-0.923)	0.55	1.525
Stance time right (ms)	781.532 ± 5.250	0.672	0.982 (0.968-0.990)	0.09	0.25
Mean pressure left (g/cm^2^)	772.883 ± 3.435	0.444	0.957 (0.925-0.976)	0.092	0.255
Mean pressure right (g/cm^2^)	774.243 ± 11.728	1.515	0.922 (0.865-0.957)	0.423	1.173
Peak pressure left (g/cm^2^)	1467.964 ± 14.614	0.996	0.927 (0.874-0.960)	0.269	0.746
Peak pressure right (g/cm^2^)	1361.396 ± 10.761	0.79	0.948 (0.910-0.971)	0.18	0.5
**10 mm of LLD**					
Stance time left (ms)	789.550 ± 3.527	0.447	0.982 (0.969-0.990)	0.06	0.166
Stance time right (ms)	785.045 ± 6.698	0.853	0.976 (0.958-0.987)	0.132	0.366
Mean pressure left (g/cm^2^)	777.108 ± 2.357	0.303	0.966 (0.942-0.981)	0.056	0.155
Mean pressure right (g/cm^2^)	733.541 ± 5.050	0.688	0.913 (0.851-0.952)	0.203	0.563
Peak pressure left (g/cm^2^)	1468.613 ± 24.001	1.634	0.930 (0.880-0.962)	0.432	1.199
Peak pressure right (g/cm^2^)	1277.036 ± 13.052	1.022	0.930 (0.879-0.961)	0.27	0.75
**15 mm of LLD**					
Stance time left (ms)	792.162 ± 2.742	0.346	0.985 (0.975-0.992)	0.042	0.118
Stance time right (ms)	806.757 ± 3.375	0.418	0.981 (0.967-0.990)	0.058	0.16
Mean pressure left (g/cm^2^)	785.369 ± 6.983	0.889	0.968 (0.944-0.982)	0.159	0.441
Mean pressure right (g/cm^2^)	728.414 ± 8.107	1.113	0.944 (0.903-0.969)	0.263	0.73
Peak pressure left (g/cm^2^)	1470.207 ± 10.147	0.69	0.945 (0.906-0.970)	0.162	0.449
Peak pressure right (g/cm^2^)	1262.550 ± 12.141	0.962	0.899 (0.826-0.945)	0.306	0.847
**20 mm of LLD**					
Stance time left (ms)	793.784 ± 3.783	0.477	0.976 (0.959-0.987)	0.074	0.205
Stance time right (ms)	807.658 ± 4.904	0.607	0.986 (0.975-0.992)	0.072	0.199
Mean pressure left (g/cm^2^)	785.207 ± 4.703	0.599	0.964 (0.937-0.980)	0.114	0.315
Mean pressure right (g/cm^2^)	724.856 ± 8.859	1.222	0.889 (0.810-0.939)	0.407	1.129
Peak pressure left (g/cm^2^)	1457.306 ± 26.924	1.848	0.882 (0.798-0.935)	0.635	1.759
Peak pressure right (g/cm^2^)	1247.450 ± 3.442	0.276	0.878 (0.790-0.933)	0.096	0.265

SD = standard deviation; CoV = coefficient of variation; ICC = intraclass correlation coefficient; 95% CI = 95 percent confidence interval; SEM% = standard error of measurement percentage; MDC% = minimum detectable change percentage; LLD = leg length discrepancy.

### Intersession reliability

The average for measurements from each test session and the ICC, CoV, SEM%, MDC%, RC% and Student’s t test for intersession reliability are presented in Table 4. The ICC for intersession reliability ranged from 0.909 to 0.990 and the SEM ranged from 0.05% to 0.49%. The CoV ranged from 0.5% to 1.70%, the MDC ranged from 0.14% to 1.36% and the RC ranged from 0.84% to 19.22%. All variables were compared using Student’s t test and had P-values > 0.05.

**Table 4.** Intersession reliability of dynamic variables for each foot under simulated LLD conditions

Variables	Mean ± SD	CoV (%)	ICC (2.1) (95% CI)	SEM%	MDC%	RC%	STS
**0 mm of LLD**							
Stance time left (ms)	777.730 ± 5.365	0.69	0.960 (0.936-0.977)	0.14	0.38	11.37	0.483
Stance time right (ms)	772.162 ± 5.318	0.69	0.969 (0.951-0.982)	0.12	0.34	9.18	0.88
Mean pressure left (g/cm^2^)	769.901 ± 11.210	1.46	0.962 (0.940-0.978)	0.28	0.79	13.17	0.115
Mean pressure right (g/cm^2^)	777.932 ± 11.472	1.47	0.946 (0.914-0.969)	0.34	0.95	15.67	0.101
Peak pressure left (g/cm^2^)	1461.203 ± 16.645	1.14	0.954 (0.926-0.973)	0.24	0.68	14.12	0.177
Peak pressure right (g/cm^2^)	1461.036 ± 22.094	1.51	0.956 (0.930-0.975)	0.32	0.88	19.22	0.196
**5 mm of LLD**							
Stance time left (ms)	780.068 ± 11.486	1.47	0.923 (0.878-0.956)	0.41	1.13	16.46	0.758
Stance time right (ms)	775.941 ± 11.347	1.46	0.936 (0.898-0.963)	0.37	1.03	13.36	0.207
Mean pressure left (g/cm^2^)	774.577 ± 5.537	0.71	0.974 (0.959-0.985)	0.12	0.32	9.00	0.566
Mean pressure right (g/cm^2^)	768.117 ± 11.903	1.55	0.953 (0.925-0.973)	0.34	0.93	13.66	0.172
Peak pressure left (g/cm^2^)	1481.595 ± 19.287	1.30	0.955 (0.928-0.974)	0.28	0.77	12.88	0.097
Peak pressure right (g/cm^2^)	1363.243 ± 12.762	0.94	0.967 (0.947-0.981)	0.17	0.47	10.11	0.751
**10 mm of LLD**							
Stance time left (ms)	788.378 ± 4.897	0.62	0.990 (0.984-0.994)	0.06	0.17	3.80	0.357
Stance time right (ms)	789.054 ± 7.086	0.90	0.986 (0.978-0.992)	0.11	0.29	6.90	0.088
Mean pressure left (g/cm^2^)	778.770 ± 4.353	0.56	0.984 (0.974-0.991)	0.07	0.20	0.84	0.427
Mean pressure right (g/cm^2^)	737.311 ± 9.678	1.31	0.961 (0.938-0.978)	0.26	0.72	8.78	0.173
Peak pressure left (g/cm^2^)	1462.455 ± 17.696	1.21	0.957 (0.932-0.975)	0.25	0.70	12.48	0.426
Peak pressure right (g/cm^2^)	1275.865 ± 18.723	1.47	0.943 (0.909-0.967)	0.35	0.97	13.69	0.874
**15 mm of LLD**							
Stance time left (ms)	789.324 ± 6.093	0.77	0.990 (0.985-0.994)	0.08	0.21	6.63	0.204
Stance time right (ms)	802.568 ± 6.811	0.85	0.987 (0.979-0.992)	0.10	0.27	8.66	0.159
Mean pressure left (g/cm^2^)	782.297 ± 5.775	0.74	0.984 (0.974-0.991)	0.09	0.26	6.91	0.184
Mean pressure right (g/cm^2^)	725.068 ± 7.155	0.99	0.964 (0.943-0.980)	0.19	0.52	11.48	0.344
Peak pressure left (g/cm^2^)	1461.068 ± 12.798	0.88	0.957 (0.932-0.976)	0.18	0.50	12.42	0.238
Peak pressure right (g/cm^2^)	1225.914 ± 18.399	1.50	0.909 (0.855-0.948)	0.45	1.25	16.40	0.382
**20 mm of LLD**							
Stance time left (ms)	791.036 ± 4.392	0.56	0.986 (0.978-0.992)	0.07	0.18	6.84	0.234
Stance time right (ms)	807.793 ± 4.042	0.50	0.990 (0.984-0.994)	0.05	0.14	5.86	0.946
Mean pressure left (g/cm^2^)	783.838 ± 4.090	0.52	0.980 (0.969-0.989)	0.07	0.20	8.96	0.645
Mean pressure right (g/cm^2^)	724.225 ± 5.874	0.81	0.948 (0.917-0.970)	0.18	0.51	10.24	0.84
Peak pressure left (g/cm^2^)	1451.995 ± 23.364	1.61	0.951 (0.922-0.972)	0.36	0.99	12.00	0.472
Peak pressure right (g/cm^2^)	1239.901 ± 21.097	1.70	0.917 (0.868-0.952)	0.49	1.36	12.67	0.259

SD = standard deviation; CoV = coefficient of variation; ICC = intraclass correlation coefficient; 95% CI = 95 percent confidence interval; SEM% = standard error of measurement percentage; MDC% = minimum detectable change percentage; RC% = repeatability coefficient percentage; STS = Student’s t test significance; LLD = leg length discrepancy.

**Figure 1.**
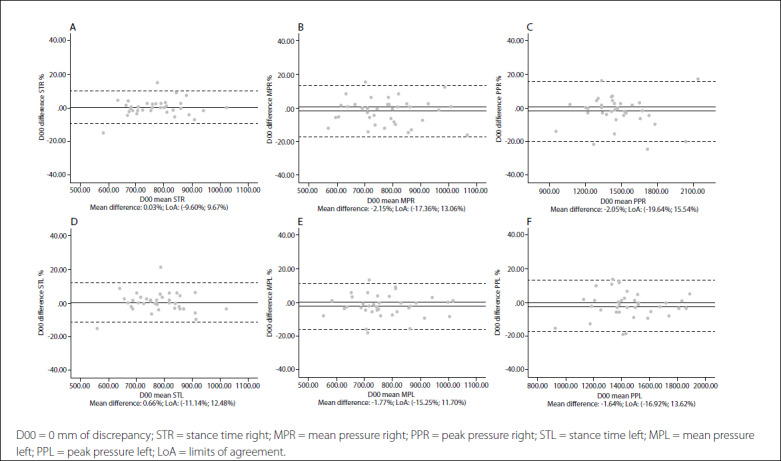
Bland-Altman plots for dynamic variables for each foot without leg length discrepancy (LLD). Differences between sessions plotted against the mean. Stance time right (A); Mean pressure right (B); Peak pressure right (C); Stance time left (D); Mean pressure left (E); Peak pressure left (F). Abbreviations: D00, 0 mm of discrepancy; STR, stance time right; MPR, mean pressure right; PPR, peak pressure right; STL, stance time left; MTL, mean pressure left; PPL, peak pressure left; LoA, limits of agreement.

**Figure 2.**
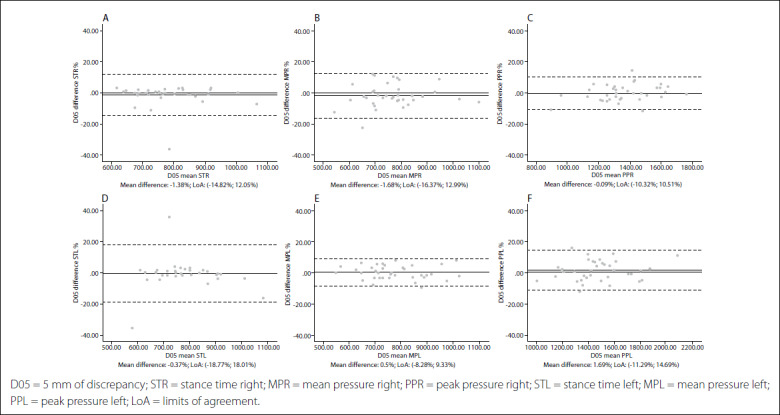
Bland-Altman plots for dynamic variables for each foot with 5 mm of leg length discrepancy (LLD). Differences between sessions plotted against the mean. Stance time right (A); Mean pressure right (B); Peak pressure right (C); Stance time left (D); Mean pressure left (E); Peak pressure left (F).

**Figure 3.**
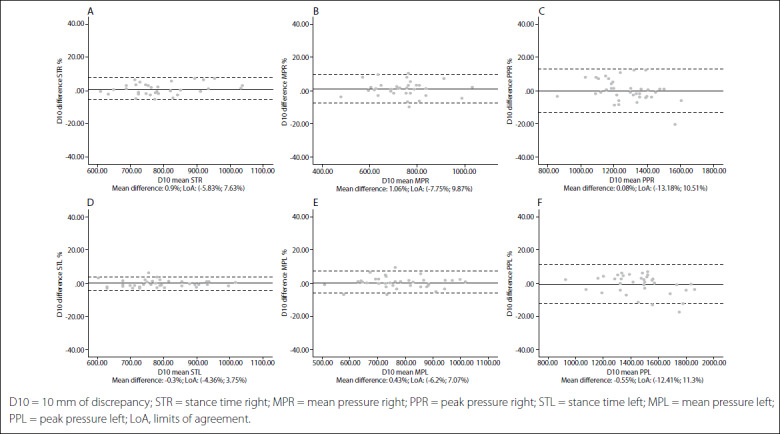
Bland-Altman plots for dynamic variables for each foot with 10 mm of leg length discrepancy (LLD). Differences between sessions plotted against the mean. Stance time right (A); Mean pressure right (B); Peak pressure right (C); Stance time left (D); Mean pressure left (E); Peak pressure left (F).

**Figure 4.**
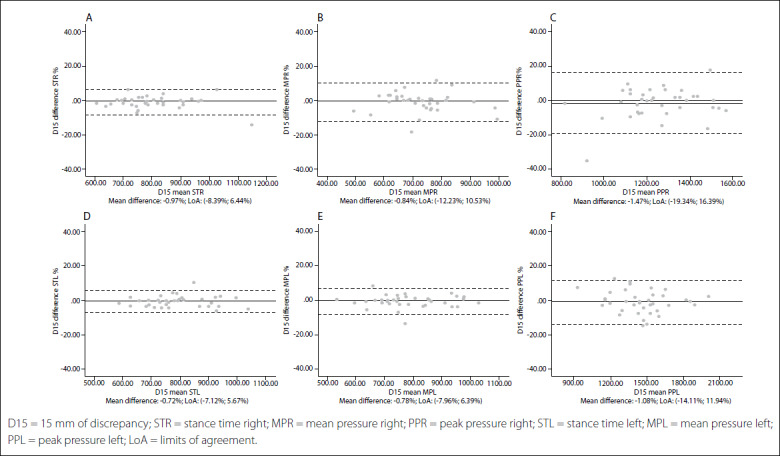
Bland-Altman plots for dynamic variables for each foot with 15 mm of leg length discrepancy (LLD). Differences between sessions plotted against the mean. Stance time right (A); Mean pressure right (B); Peak pressure right (C); Stance time left (D); Mean pressure left (E); Peak pressure left (F).

**Figure 5.**
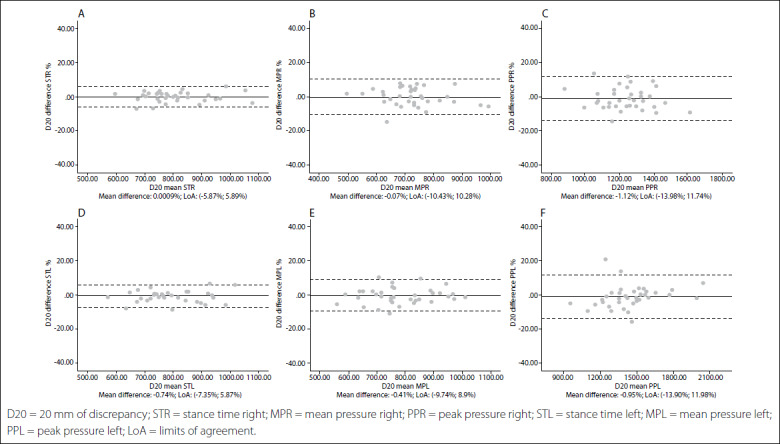
Bland-Altman plots for dynamic variables for each foot with 20 mm of leg length discrepancy (LLD). Differences between sessions plotted against the mean. Stance time right (A); Mean pressure right (B); Peak pressure right (C); Stance time left (D); Mean pressure left (E); Peak pressure left (F).

Intersession limits of agreement and Bland-Altman plots are shown in Figures 1,2,3,4,5. The mean differences between sessions for the variables studied ranged from -2.15% to 1.69%.

## DISCUSSION

Plantar pressure platforms are clinical devices that are used to assess foot interactions with the ground,^21^ and to screen gait patterns that could lead to pathological conditions.^22^ The Podoprint platform system is commonly used by clinicians and researchers as a tool to develop their work. One of the applications of this technology could be to study LLD and its consequences on the patient’s health.^28^ Hence, the aim of this study was to determine the intra and intersession repeatability and reliability of dynamic parameters of this pressure platform among subjects with simulated leg length discrepancy.

The ICC is an extensively used descriptive statistic for quantifying the repeatability of a measurement. Using the classification proposed by Landis and Koch, ICC values between 0.40 and 0.60 have moderate reliability, whereas scores in the highest category, ranging from 0.80 to 1.00, are considered nearly perfect.^35^ Other authors have indicated that an ICC value of at least 0.75^36^ needs to be available to obtain reliability. According to Portney and Watkins’ recommendations, clinical measurements with an ICC greater than 0.90 improve the probability of valid measurements.^37^ In our study, the results showed consistent intra and intersession reliability and repeatability of the Podoprint platform under all LLD conditions. Intrasession variability was very low. All ICCs were higher than 0.775 and most were higher than 0.9.

SEM is considered to represent the amount of measurement error of a test and MDC shows the value for the smallest change needed in order to recognize that the observed change is real and not a result from instrument measurement error.^41^^,^^42^ In this study, the SEMs were in most of the cases lower than 1% and the MDC ranged from 0.038% and 3.037%. Low MDC values strengthened the ICCs. These results indicated that the reliability measurements were higher than those in other similar studies.^22^^,^^43^ One explanation for this may be that the measurements were obtained using the multiple dynamic mode of the platform. In this mode, the platform software automatically provides the average results from four measurements for each foot.

According to Becerro de Bengoa et al., the expected physiological changes in muscle activity, posture and gait velocity can affect variables during the measurements. Therefore, using a single trial to obtain foot dynamic parameters from a sample is not sufficient. By averaging multiple trials, the variability of gait patterns is decreased.^22^ Other authors have suggested that three trials are sufficient to obtain a consistent outcome.^44^ In our three-trial protocol, each trial needed four measurements from each foot. In fact, this is equivalent to 12 trials per session.

Gurney et al. conducted a study on the between-day reliability of repeated plantar pressure distribution measurements in a healthy population by analyzing 10-foot areas. Their intersession ICCs for total area averages were higher than 0.8 and the highest CoV was 13%.^44^ Izquierdo-Renau et al. also found ICC values greater than 0.89.^44^ In the present study, intersession repeatability was extremely high. All ICCs were greater than 0.9 for all measured dynamic variables and the highest CoV was 1.7%, which was concordant with the outcomes from previous studies.^22^^,^^25^^,^^43^ We did not find any significant differences between the first and second sessions when comparing means, with P-values > 0.05. All of the variables evaluated were highly homogeneous across all LLD conditions and for both feet.

In addition, the limits of agreement and Bland-Altman plots were also calculated and showed differences between sessions plotted against the mean.^38^ This complementary analysis revealed that the distribution of such graphs did not show any systematic measurement errors or heteroscedasticity. All of the values for the variables were similar, independent of the LLD condition, and also presented very high repeatability and reliability in all cases.

One limitation of the present study was that pressures were evaluated for total foot plantar surface. Previous investigations have found greater intrasession variability when using a regional analysis.^25^^,^^45^ Furthermore, the lifts were used in non-randomized order and, thus, the potential learning effects may have influenced the outcomes. Future studies should consider the relationship between several dynamic parameters measured on pressure platforms, such as total stance time, mean pressure, peak pressure and degree of simulated LLD. In addition, patients who have skeletal malalignment disorders such as patellofemoral or hip disorders should be dynamically evaluated using this plantar pressures device under conditions of leg length discrepancy.^10^

## CONCLUSIONS

The results from this study indicate that the Podoprint platform is a reliable system for assessing dynamic parameters for both normal and simulated LLD subjects, due to its high reliability, independent of the LLD condition. This testing device can be confidently used by researchers and clinicians to assess dynamic parameters during research or clinical screenings. Given the reliability results from this study, future studies should evaluate plantar pressures under conditions of length leg discrepancy that are associated with skeletal malalignment disorders such as patellofemoral disorders.
